# UBE2C Overexpression Aggravates Patient Outcome by Promoting Estrogen-Dependent/Independent Cell Proliferation in Early Hormone Receptor-Positive and HER2-Negative Breast Cancer

**DOI:** 10.3389/fonc.2019.01574

**Published:** 2020-01-23

**Authors:** Yu-Jin Kim, Gyunghwa Lee, Jinil Han, Kyoung Song, Joon-Seok Choi, Yoon-La Choi, Young Kee Shin

**Affiliations:** ^1^Laboratory of Cancer Genomics and Molecular Pathology, Samsung Medical Center, Sungkyunkwan University School of Medicine, Seoul, South Korea; ^2^Laboratory of Molecular Pathology and Cancer Genomics, Department of Pharmacy, College of Pharmacy, Seoul National University, Seoul, South Korea; ^3^Gencurix, Inc., Seoul, South Korea; ^4^The Center for Companion Diagnostics, LOGONE Bio Convergence Research Foundation, Seoul, South Korea; ^5^College of Pharmacy, Daegu Catholic University, Gyeongsan-si, South Korea; ^6^Department of Pathology and Translational Genomics, Samsung Medical Center, Sungkyunkwan University School of Medicine, Seoul, South Korea; ^7^Department of Health Sciences and Technology, SAIHST, Sungkyunkwan University, Seoul, South Korea; ^8^The Center for Anti-cancer Companion Diagnostics, BioMAX/N-Bio, Seoul National University, Seoul, South Korea; ^9^Department of Molecular Medicine and Biopharmaceutical Sciences, Graduate School of Convergence Science and Technology, Seoul National University, Seoul, South Korea

**Keywords:** UBE2C, HR+/HER2– breast cancer, estrogen, cell cycle, tamoxifen, apoptosis

## Abstract

We previously showed that *UBE2C* mRNA expression is significantly associated with poor prognosis only in patients with hormone receptor (HR)+/human epidermal growth factor receptor 2 (HER2)– breast cancer. In this study, we further reanalyzed the correlation between *UBE2C* mRNA expression and clinical outcomes in patients with HR+/HER2– breast cancer, and we investigated the molecular mechanism underlying the role of UBE2C modulation in disease progression in this subgroup of patients. Univariate and multivariate analyses showed that high *UBE2C* expression was associated with significantly shorter survival of breast cancer patients with pN0 and pN1 tumors but not pN2/N3 tumors (*P* < 0.05). *In vitro* functional experiments in HR+/HER2– breast cancer cells showed that UBE2C expression is a tumorigenic factor, and that estrogen upregulated *UBE2C* mRNA and protein by directly binding to the *UBE2C* promoter region. UBE2C knockdown inhibited cell proliferation by affecting cell cycle progression, and UBE2C overexpression was associated with estrogen-independent growth. UBE2C depletion markedly increased the cytotoxicity of tamoxifen by inducing apoptosis. The present findings suggest that UBE2C overexpression is correlated with relapse and promotes estrogen-dependent/independent proliferation in early HR+/HER2– breast cancer.

## Introduction

Breast cancer is classified according to estrogen receptor (ER), progesterone receptor (PR), and human epidermal growth factor receptor 2 (HER2) expression status into hormone receptor HR+/HER2– (ER+ or PR+/HER2–), HR+/HER2+ (ER+ or PR+/HER2+), HR–/HER2+ (ER–/PR–/HER2+), or triple-negative breast cancer (TNBC; ER–/PR–/HER2–); the HR+/HER2– subtype accounts for 25–40% of breast cancers (1, 2). Above 70% of patients with HR+/HER2– breast cancer remain disease-free without chemotherapy after surgery. However, more than 5% of patients with HR+/HER2– breast cancer have a higher of late recurrence beyond 5 years after primary adjuvant hormone therapy than those with other subtypes (3–5). Therefore, an exact estimation of the risk of recurrence could be useful for decision-making regarding the duration of adjuvant hormone therapy or treatment with adjuvant chemotherapy.

Because gene expression provides significant prognostic or predictive information for patients with HR+ breast cancer, several commercial assays such as Oncotype DX, MammaPrint, and EndoPredict, which are based on the expression of multiple genes in frozen or formalin-fixed, paraffin-embedded (FFPE) samples, have been developed (6–8). These assays predict the risk of distant recurrence after hormone therapy and help to identify patients who may benefit from adjuvant chemotherapy by discriminating between high- and low-risk patients with early HR+ breast cancer. However, these assays were validated in only Western countries, therefore the prognostic value of these signatures in Asian breast cancer patients remains unclear as well as Korean. In a previous study, we identified 384 candidate prognostic genes associated with distant metastasis in patients with lymph node (LN) negative early breast cancer (9). Of these 384 genes, 16 candidate prognostic genes were selected, and the association between the expression of these genes determined by quantitative real-time reverse transcription-PCR (qRT-PCR) and clinical outcomes was evaluated in 997 FFPE tissue samples from 819 patients with breast cancer (9, 10). Six genes were selected to develop a prognostic model for predicting the risk of distant recurrence or distant metastasis, and the performance of the model was compared with that of classical clinicopathological risk factors (10–13).

One of the six genes included in the model is ubiquitin-conjugating enzyme E2C (UBE2C), which plays an important role in the ubiquitin-proteasome system. The ubiquitin-proteasome system precisely regulates the cell cycle through proteasome-mediated protein degradation pathways in eukaryotes. Dysregulation of ubiquitin-dependent processes is involved in the occurrence and progression of cancer, and the ubiquitin-proteasome system has thus become a new druggable target in certain cancers such as bladder carcinoma, cervical cancer, colorectal carcinoma, esophageal squamous cell carcinoma, lung cancer, and pancreatic carcinoma (14–20). High UBE2C expression is associated with a high degree of malignancy, low differentiation, high metastatic tendency, and poor patient survival in a wide range of solid tumors including breast cancer (21–25). We previously reported that UBE2C is significantly associated with poor prognosis in patients with HR+/HER2– breast cancer (9, 10). Consistent with our report, Filipits et al. reported the use of *UBE2C* mRNA expression as a marker in the EndoPredict assay for predicting the risk of recurrence or distant metastasis in patients with HR+/HER2– breast cancer (8). However, the clinical and functional significance of UBE2C expression in HR+/HER2– breast cancer remains unknown.

In this study, we examined the correlation between *UBE2C* mRNA expression and clinical outcomes in patients with HR+/HER2– breast cancer. We also evaluated the expression status of UBE2C and investigated the molecular mechanism underlying the role of UBE2C regulation in HR+/HER2– breast cancer progression.

## Materials and Methods

### Patient Samples

A total of 997 FFPE tissue specimens were obtained from patients with breast cancer who underwent curative resection of primary tumors with LN dissection at Samsung Medical Center (SMC, Korea) between 1994 and 2002. The protocol for the present study was approved by the SMC Institutional Review Board (IRB file No. 2008-12-035). Tumor size and LN involvement were evaluated according to the American Joint Committee on Cancer 7th TNM Staging System, and tumor histological grades were determined according to the Bloom–Richardson grading scheme. Paraffin-embedded tissue samples (mounted on slides) were analyzed to define tumor regions and select representative tumor areas for further analysis. Breast cancer specimens were classified into subtypes using an immunohistochemical assay with ER, PR, and HER2 as markers.

### qRT-PCR Analysis of Patient Samples

RNA was isolated from patient-derived FFPE samples using a tissue preparation system (Siemens AG), and qRT-PCR was performed to measure the expression levels of *UBE2C* (Roche Applied Science). The results of qRT-PCR were expressed as cycle threshold (Ct) values. The Ct value for *UBE2C* was normalized to a relative expression value (ΔCt value) using three reference genes (*CTBP1, CUL1*, and *UBQLN1*), and it was calculated as follows:

$$\begin{array}{l} \Delta \text{Ct}\_\text{target}=\;[(\text{Ct}\_\text{CTBP1 + Ct}\_\text{CUL1 + Ct}\_\text{UBQLN1})\text{/3}]\\ \;-\text{Ct}\_\text{target + 30}. \end{array}$$

### Statistical Analysis

Expression values were compared using the independent *t*-test and Chi-square test. Univariate and multivariate survival analyses were performed using the Cox proportional hazard model. Study endpoints were disease-free survival (DFS, time from primary surgery to any first event of recurrence), distant metastasis-free survival (DMFS, time from primary surgery until distant metastasis), and overall survival (OS, time from primary surgery until death from any cause). The genomic and clinical variables that were significant in the univariate analysis were included in the multivariate analysis to identify independent predictors of DFS, DMFS, and OS. Survival rates were estimated by the Kaplan–Meier (KM) method, and the log-rank test was used for assessing the difference in survival among patient groups. To classify patients into low- and high-expression groups for distant metastasis, the cut-off value was set as the value that maximized the sum of sensitivity and specificity. Statistical significance was considered at *P* < 0.05. All statistical analyses were performed using R 3.5.1 (http://r-project.org).

### Cell Culture

The human breast cell lines were obtained from the American Type Culture Collection and Korean Cell Line Bank. All cell lines were cultured according to the manufacturers' recommendations_._ Cell lines were validated by human cell line authentication (STR DNA profiling) using the AmpFLSTR™ Identifiler PCR Amplification Kit (Thermo Fisher Scientific).

### Real-Time qRT-PCR in Cells

The expression levels of *UBE2C* mRNA were measured by real-time qRT-PCR. Total RNA was isolated using RiboEx (GeneAll) and the Hybrid-R kit (GeneAll) followed by the Transcriptor First Strand cDNA Synthesis Kit (Roche Applied Science), according to the manufacturers' instructions. qRT-PCR was performed on cDNA using a LightCycler 480 System (Roche Applied Science). The UBE2C primers used were as follows: 5′-TGCCGAGCTCTGGAAAAA-3′ (forward primer) and 5′-AAAAGACGACACAAGGACAGG-3′ (reverse primer). The amplified *UBE2C* cDNAs obtained using these primers consisted of five transcript isoforms among seven coding sequence (CDS) transcripts (https://www.ensembl.org). The HPRT primers were used as a control. Commercial Universal Probe Library (UPL) probes were purchased from Roche Applied Science.

### Western Blot Analysis

Cells were lysed with RIPA buffer [20 mM Tris-HCl (pH 8.0), 150 mM NaCl, 10% glycerol, 1% NP40, and 2 mM EDTA]. Equal amounts of protein were subjected to 10% SDS-PAGE and transferred to a nitrocellulose membrane (Millipore). The membrane was incubated overnight at 4°C with primary anti-UBE2C (Abcam, ab187181), anti-cleaved PARP (Cell Signaling, #9541), anti-cleaved CASPASE 3 (Cell Signaling, #9661), anti-ERα (Santa Cruz Biotechnology, sc-8002), or anti-β-ACTIN (Santa Cruz Biotechnology, sc-47778) antibody, and then washed for 30 min with TBST. The membrane was then incubated for 1 h with horseradish peroxidase-conjugated secondary goat anti-rabbit or anti-mouse antibody (diluted 1:1,000 in 5% non-fat milk) before washing for 30 min with TBST. Signals were detected using an ECL solution (Promega).

### Cloning and siRNA Knockdown

A *UBE2C* cDNA expression construct was generated by amplifying the region encoding the primary transcript from human *UBE2C* mRNA (NM_007019.4). The PCR products were cloned into the N-terminal pEF/HIS A vector (Addgene). The full sequence of wild-type *UBE2C* cDNA was confirmed by Sanger sequencing. *UBE2C* mRNA knockdown was performed using On-TARGET plus Human UBE2C specific siRNA (Dharmacon). Four target sequences of *UBE2C* were used for mixed siRNA.

### Cell Viability, Proliferation, and Migration Assays

Cell viability was assessed using the live cell imaging tool (IncuCyte ZOOM, Essen BioScience). For proliferation assays using estrogen, equal numbers of cells were plated in 12-well plates and treated with 17-beta-estradiol (E2) added to the media at 10 nM every 48 h after siRNA treatment. The total number of cells was counted. The migration of MCF-7 and T47D cells was analyzed using Transwell chambers (Corning) and the wound healing assay in T47D cells. Treated and control cells were serum starved for 24 h and plated in equal numbers in Transwell chambers. After 24 h of incubation, cells that migrated to the bottom surface were detached and lysed using a cell detachment solution and 4× cell lysis buffer (Millipore). The cell lysate was incubated with CyQuant GR Dye 1 (Millipore), and fluorescence was measured at 485/535 nm. Cell migration was assessed using the wound healing assay in T47D cells. Equal numbers of treated and control cells were cultured to 90–95% confluency in 96-well plates. Cell monolayers were wounded using a sterilized wound maker (Cell player), and gap recovery was measured using live cell imaging (IncuCyte ZOOM, Essen BioScience).

### Clonogenic Assay in Two-Dimensions (2D) and Three-Dimensions (3D), Apoptosis Assay, and Cell Cycle Distribution Analysis

For the clonogenic assay in 2D, cell numbers were assessed indirectly by staining with crystal violet because the total amount of cell-bound dye is proportional to cell number. Equal numbers of treated and control cells were seeded in six-well plates at a density of 5,000 and 10,000 cells per well in medium supplemented with 10% FBS and 1% antibiotics for 14 days. Viable cells were stained with 0.5% crystal violet dye in 25% methanol for 5 min. After staining, the fixed cells were washed with phosphate-buffered saline and photographed. The dye was eluted with 10% acetic acid, and absorbance was measured at 570 nm. For the clonogenic assay in 3D, cells were cultured in Matrigel (BD Science). The bottom layer of each well was coated with 45 μL of Matrigel and allowed to form a gel by incubation for 30 min at 37°C. Then, 20,000 cells resuspended in 200 μL of matrigel were loaded onto the bottom layer of each well. Complete medium was then overlaid onto the gel and replaced every 2 days. The images of cell colonies were obtained after culturing for 7 days. Cell apoptosis was determined using the FITC Annexin V Apoptosis Detection Kit I (BD Biosciences) following treatment with 1 μM staurosporine (STS, Sigma-Aldrich). Cell apoptosis was analyzed with a FACSAria system (BD Biosciences). Harvested cells were stained with propidium iodide (PI) and subjected to flow cytometric analysis (BD FACSAria).

### Chou–Talalay Analysis

MCF-7 cells were treated with siRNA against UBE2C and tamoxifen, and growth inhibition was measured using the WST assay. MCF-7 cells were seeded into six-well plates and treated with tamoxifen at 48 h after siRNA transfection. The pharmacological interaction between UBE2C-specific siRNA and tamoxifen was assessed by Chou–Talalay analysis (26, 27). This method assesses synergy and antagonism by quantifying the divergence of the combination effect from the expected additive effect of the two therapeutic agents. A combination index (CI) was estimated from the dose-effect data. A CI of <1, =1, and >1 indicates synergy, additive effects, and antagonism, respectively.

### Chromatin Immunoprecipitation (ChIP)-PCR

Cells were plated in a 150 mm dish at 80–90% density, and chromatin immunoprecipitation (ChIP) was performed using an EZ ChIP kit (Millipore) as described in the instruction manual. In brief, cells were fixed with 10 ml formaldehyde (37%, Sigma) for 10 min to crosslink proteins to DNA. Cell suspensions were then sonicated (Bioruptor), and the chromatin was sheared to a DNA size of ~200–1,000 base pairs. The cross-linked protein/DNA complexes were subjected to immunoprecipitation with anti-ERα (Santa Cruz Biotechnology, sc-8002) and normal mouse IgG as the negative control. Protein/DNA complexes were purified using a spin column. Following purification of associated DNA, semi-quantitative PCR was performed to quantify the DNA sequence associated with ERα using specific *UBE2C* promoter primers. The primer sequences used for ChIP-PCR assay were as follows (Fwd: forward, Rev: reverse): *GREB1* (positive control, Fwd: 5′-CAGCTGACTGTCTTCCACCA-3′, Rev: 5′-CCACCGTTTCGTGTCTTCTT-3′); *GAPDH* (negative control, Fwd: 5′-TACTAGCGGTTTTACGGGCG-3′, Rev: 5′-TCGAACAGGAGGAGCAGAGAGCGA-3′) (28); and *UBE2C* (Fwd: 5′-ACCAATCGGTTGTCAGAAGC-3′, Rev: 5′-GGCAGAGAGACAGGAACTCG-3′).

## Results

### Implications of *UBE2C* mRNA Expression in the Survival of Patients With HR+/HER2– Breast Cancer

The clinical significance of UBE2C in breast cancer was investigated by analyzing *UBE2C* mRNA expression in invasive breast cancer tissues. In a previous study, we performed subgroup analysis according to molecular breast cancer subtype using FFPE samples from 819 breast cancer patients. The results of univariate analysis showed that *UBE2C* mRNA expression as a continuous variable is statistically significantly associated with DFS, DMFS, and OS in only patients with HR+/HER2– breast cancer regardless of LN negativity or positivity (10). Of the previous cohort, 410 patients with HR+/HER2– breast cancer were included in this study; the detailed clinical characteristics of patients were shown in Table 1. Briefly, the median patient age was 47.3 years (range, 25.2–80.5), and 49.5% (203/410) were LN negative. Of 410 patients, 342 (83.4%) received adjuvant chemotherapy, and histologic grade 2 was predominant in the patient group (48.5%, 199/410).

**Table 1:** Clinical characteristics of the breast cancer patients in this study.

	**HR+/HER2– (*n* = 410) No. (%)**
**Median age (min–max) (years)**	47.3 (25.2–80.5)
**Age (years)**	
<50	241 (58.8%)
≥50	169 (41.2%)
**Tumor size (cm)**	
≤ 2	183 (44.6%)
2-5	204 (49.8%)
>5	23 (5.6%)
**Lymph node status**	
Negative	203 (49.5%)
Positive	207 (50.5%)
**pN**	
0	203 (49.5%)
1	112 (27.3%)
2	51 (12.4%)
3	44 (10.7%)
**Pathologic stage**	
I	113 (27.6%)
II	197 (48.0%)
III	100 (24.4%)
**Histologic grade**	
1	77 (18.8%)
2	199 (48.5%)
3	123 (30%)
Unknown	11 (2.7%)
**Nuclear grade**	
1	60 (14.6%)
2	256 (62.4%)
3	81 (19.8%)
Unknown	13 (3.2%)
**Hormone therapy**	
No	21 (5.1%)
Yes	379 (92.4%)
Unknown	10 (2.4%)
**Chemotherapy**	
No	68 (16.6%)
Yes	342 (83.4%)
Unknown	0 (0.0%)
**Radiotherapy**	
No	162 (39.5%)
Yes	247 (60.2%)
Unknown	1 (0.2%)
**NPI**	
1	113 (27.6%)
2	113 (27.6%)
3	113 (27.6%)
4	58 (14.1%)
Unknown	13 (3.2%)

*HR, hormone receptor; HER2, human epidermal growth factor receptor 2; TNBC, triple-negative breast cancer; pN, pathologic nodal status; NPI, Nottingham prognostic index*.

**Figure 1:**
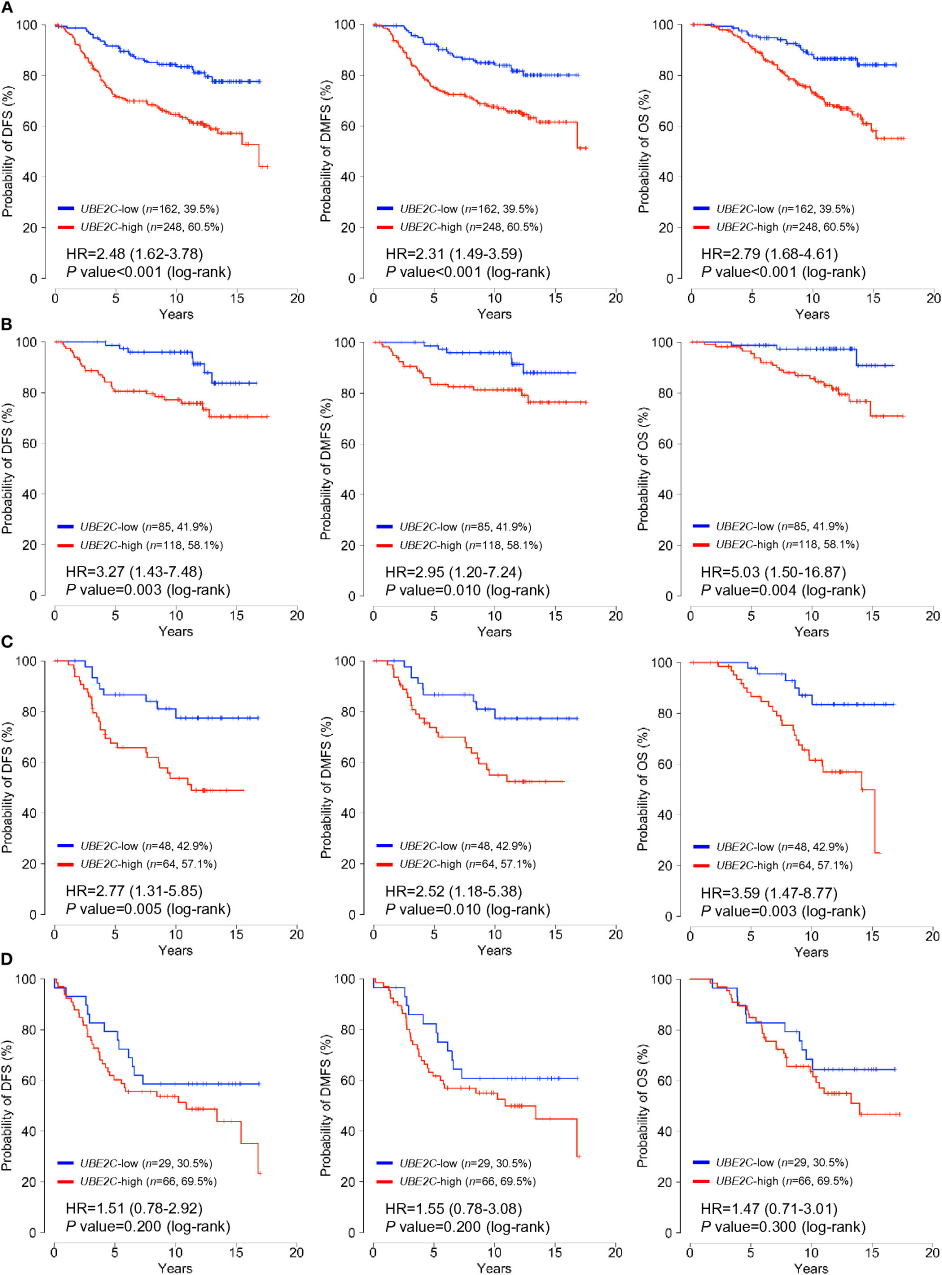
*UBE2C* mRNA expression is strongly associated with poor survival in patients with HR+/HER2– breast cancer. The impact of *UBE2C* mRNA expression on DFS (left panels), DMFS (middle panels), and OS (right panels) of **(A)** all, **(B)** pN0, **(C)** pN1, or **(D)** pN2/N3 patients with HR+/HER2**–** breast cancer was analyzed using the Kaplan–Meier method.

Patients were classified into low- or high-UBE2C groups according to a cut-off for normalized expression of *UBE2C* mRNA of 29.05; 60.5% (*n* = 248) were assigned to the high-expression group and the remaining patients were classified as the low-expression group. The association of classical clinicopathological factors and *UBE2C* gene expression levels with clinical outcomes was analyzed. Univariate analysis of DFS, DMFS, and OS indicated that positive LN involvement, large tumor size, high histologic grade, and high *UBE2C* expression were significantly associated with poor survival in all patients (*P* < 0.05, Supplementary Table 1). However, univariate, Kaplan–Meier, and multivariate analyses of subgroups according to LN status revealed that high *UBE2C* expression was associated with significantly shorter DFS, DMFS, and OS in breast cancer with pN0 and pN1 tumors but not in pN2/N3 tumors (*P* < 0.05, Supplementary Table 1, Figure 1, and Table 2). The results indicate that high *UBE2C* expression has an unfavorable impact on DFS, DMFS, and OS only in patients with HR+/HER2– pN0 and pN1 breast cancer.

**Table 2:** Multivariate analysis of the effect of *UBE2C* mRNA expression on disease-free survival (DFS), distant metastasis-free survival (DMFS), and overall survival (OS) in patients with HR+/HER2– breast cancer.

		**DFS**			**DMFS**			**OS**		
		**Hazard ratio**	**95% C.I**.	***P*-value**	**Hazard ratio**	**95% C.I**.	***P*-value**	**Hazard ratio**	**95% C.I**.	***P*-value**
**All**										
Gene expression										
*UBE2C*	(Low vs. high)	2.35	1.51–3.66	**<0.001**	2.19	1.38-3.49	**<0.001**	2.58	1.53–4.34	**<0.001**
Clinical variable										
ER (IHC)	(pN0 vs. pN1)	2.15	1.35–3.41	**0.001**	2.46	1.49–4.04	**<0.001**	2.59	1.51–4.45	**<0.001**
	(pN0 vs. pN2&3)	2.59	1.61–4.19	**<0.001**	3.00	1.79–5.02	**<0.001**	2.95	1.70–5.11	**<0.001**
Tumor size	(≤2 cm vs. >2 cm)	1.22	0.81–1.85	0.338	1.15	0.74–1.78	0.543	1.11	0.70–1.76	0.660
Histologic grade	(I&II vs. III)	1.20	0.82–1.77	0.351	1.26	0.84–1.90	0.262	1.52	0.99–2.32	0.053
**pN0**										
Gene expression										
*UBE2C*	(Low vs. high)	3.26	1.41–7.54	**0.006**	2.76	1.11–6.85	**0.029**	4.32	1.27–14.68	**0.019**
Clinical variable										
Tumor size	(≤2 cm vs. >2 cm)	1.39	0.71–2.74	0.336	1.30	0.62–2.74	0.486	1.64	0.72–3.74	0.238
Histologic grade	(I&II vs. III)	0.93	0.43–1.98	0.845	1.34	0.61–2.95	0.469	1.96	0.86–4.46	0.108
**pN1**										
Gene expression										
*UBE2C*	(Low vs. high)	2.75	1.30–5.84	**0.008**	2.53	1.18–5.42	**0.017**	3.67	1.50–9.00	**0.005**
Clinical variable										
Tumor size	(≤2 cm vs. >2 cm)	1.24	0.64–2.43	0.525	1.16	0.58–2.32	0.683	0.76	0.37–1.55	0.447
Histologic grade	(I&II vs. III)	1.03	0.53–2.04	0.921	0.97	0.47–1.98	0.928	1.11	0.52–2.35	0.786
**pN2/N3**										
Gene expression										
*UBE2C*	(Low vs. high)	1.44	0.70–2.97	0.319	1.49	0.70–3.19	0.300	1.31	0.61–2.82	0.492
Clinical variable										
Tumor size	(≤2 cm vs. >2 cm)	0.99	0.44–2.24	0.980	0.87	0.38–1.99	0.744	1.08	0.44–2.61	0.871
Histologic grade	(I&II vs. III)	1.54	0.82–2.87	0.176	1.46	0.76–2.79	0.254	1.65	0.84–3.24	0.148

*pN, lymph node status; CI, confidence interval*.

*Bold indicate significant values*.

### UBE2C Expression Status in HR+/HER2– Breast Cancer Tissues and Cell Lines

**Figure 2:**
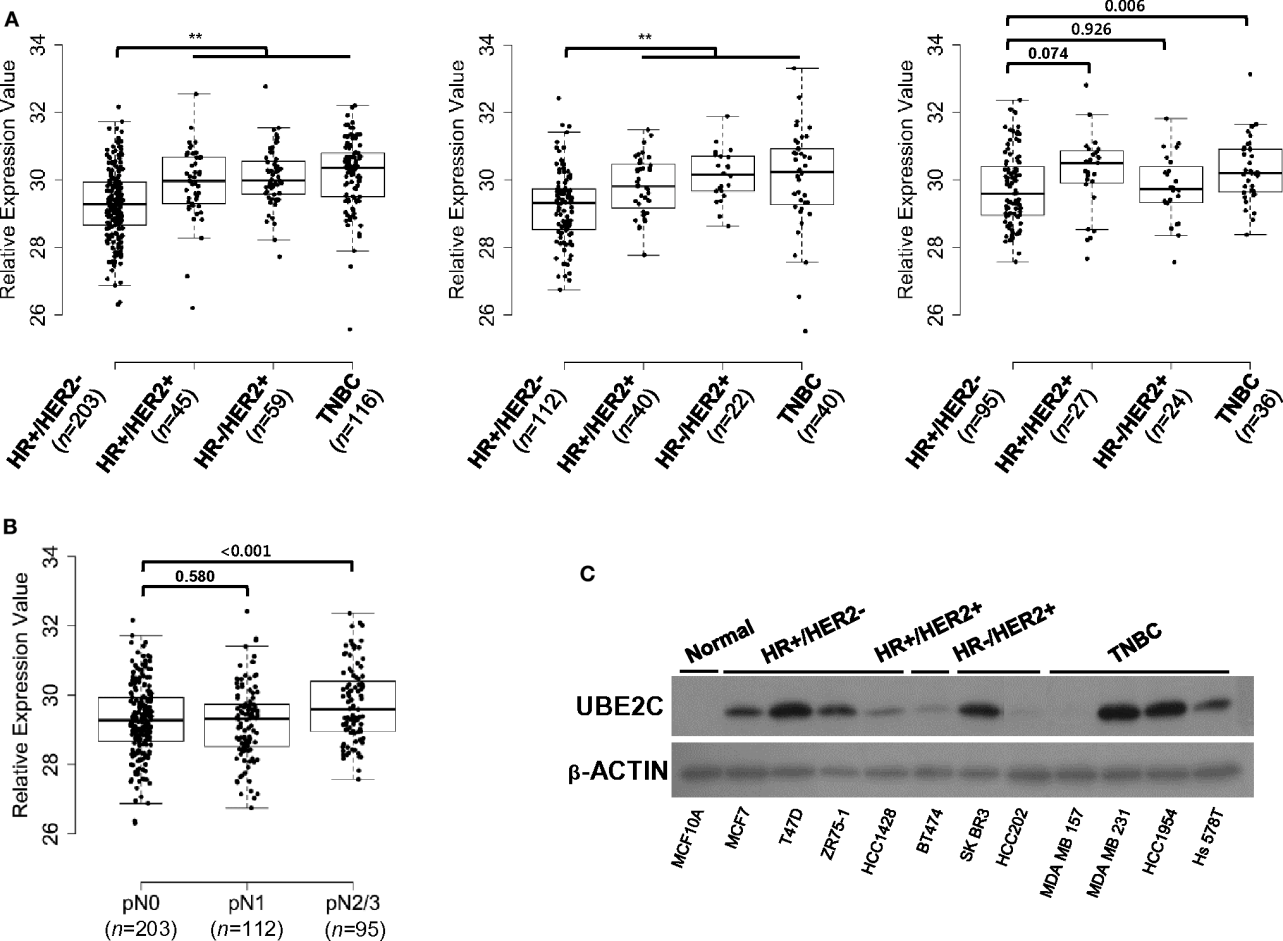
UBE2C is expressed in HR+/HER2– breast cancer tissues and cell lines. **(A)** *UBE2C* mRNA expression in FFPE breast cancer tissue was evaluated in a dataset of 819 samples from our cohort according to LN status, pN0 (left panels), pN1 (middle panel), and pN2/N3 (right panel). **(B)** *UBE2C* mRNA expression in FFPE breast cancer tissue was evaluated in a dataset of 410 samples from patients with HR+/HER2– breast cancer according to LN status, pN0, pN1, and pN2/N3. The bar indicates the median value. **(C)** UBE2C protein expression was quantified by immunoblotting in breast cancer cell lines. *P < 0.05, **P < 0.01, Student's *t*-test.

On the basis of the association between *UBE2C* expression and clinical outcomes in HR+/HER2– breast cancer, we examined the expression status of UBE2C in different breast cancer subtypes. The mRNA expression levels of *UBE2C* in four breast cancer subtypes were compared using 819 FFPE tissues from patients with different LN status. The median *UBE2C* mRNA expression was lower in HR+/HER2– breast cancers with pN0 and pN1 tumors than in other subtypes (Figure 2A). However, *UBE2C* mRNA expression in pN2/N3 tumors did not differ between HR+/HER2– and HR+/HER2+ or HR–/HER2+ subtypes excluding TNBC (Figure 2A). Among patients with HR+/HER2– breast cancer, *UBE2C* expression did not differ between pN0 and pN1 tumors, whereas it was higher in pN2/N3 tumors than in pN0 and pN1 tumors (Figure 2B). HR+/HER2– breast cancer cell lines expressed the UBE2C protein at detectable levels (Figure 2C).

### Effects of UBE2C Depletion or Overexpression on the Tumorigenicity of HR+/HER2– Breast Cancer Cells

**Figure 3:**
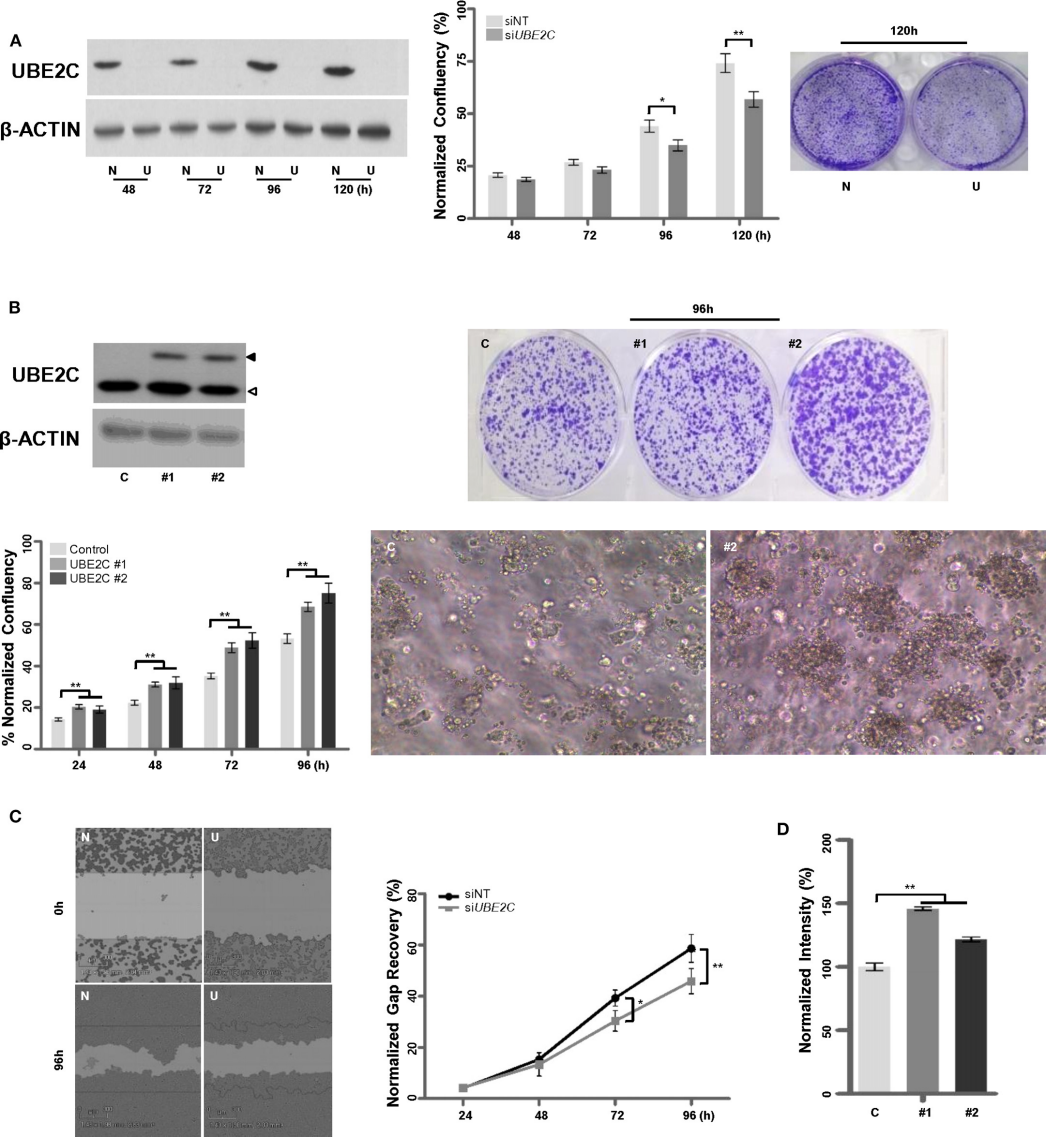
UBE2C contributes to the tumorigenicity of HR+/HER2– breast cancer cells. **(A)** The efficacy of siRNA-mediated UBE2C knockdown was evaluated by immunoblotting in T47D cells (left panel). NT, non-targeting; N, siNT; U, si*UBE2C*. The effects of UBE2C knockdown on cell proliferation were evaluated using an IncuCyte analyzer (middle panel) and clonogenic assay (right panel). **(B)** Ectopic UBE2C overexpression was confirmed by immunoblotting in MCF-7 cells (upper left panel). The effects of UBE2C overexpression on cell proliferation were evaluated using an IncuCyte analyzer (lower left panel) and clonogenic assay in 2D (upper right panel) and 3D Matrigel (100×, lower right panel). **(C)** The effects of UBE2C knockdown on cell migration were evaluated with the wound healing assay using the IncuCyte analyzer in T47D cells. **(D)** The effects of ectopic UBE2C overexpression on cell migration were evaluated with the Boyden chamber assay in MCF-7 cells. *n* = 3; Bars, SE; *P < 0.05, **P < 0.01, Student's *t*-test.

To examine whether UBE2C regulates the growth of HR+/HER2– breast cancer cells, we used T47D cells: a HR+/HER2– breast cancer cell line with high UBE2C expression level. T47D cells were transfected with *UBE2C* siRNA, and UBE2C knockdown was confirmed by immunoblotting (Figure 3A, left panel). Loss of UBE2C expression decreased cell proliferation (Figure 3A, middle and right panels) and migration (Figure 3C). Next, an HR+/HER2– cell line with moderate UBE2C expression level, MCF-7, was transfected with HA (Hemagglutinin, control) or UBE2C-expressing cDNA, and UBE2C overexpression was confirmed by immunoblotting (Figure 3B, upper left panel). Overexpression of UBE2C increased cell proliferation in 2D (Figure 3B, lower left and upper right panels) and 3D (Figure 3B, lower right panel) cell cultures and increased migration (Figure 3D). These findings indicate that UBE2C expression contributes to the tumorigenicity of HR+/HER2– breast cancer cells.

### The Growth of MCF-7 Cells Is Regulated by UBE2C Expression in an Estrogen-Dependent/Independent Manner

**Figure 4:**
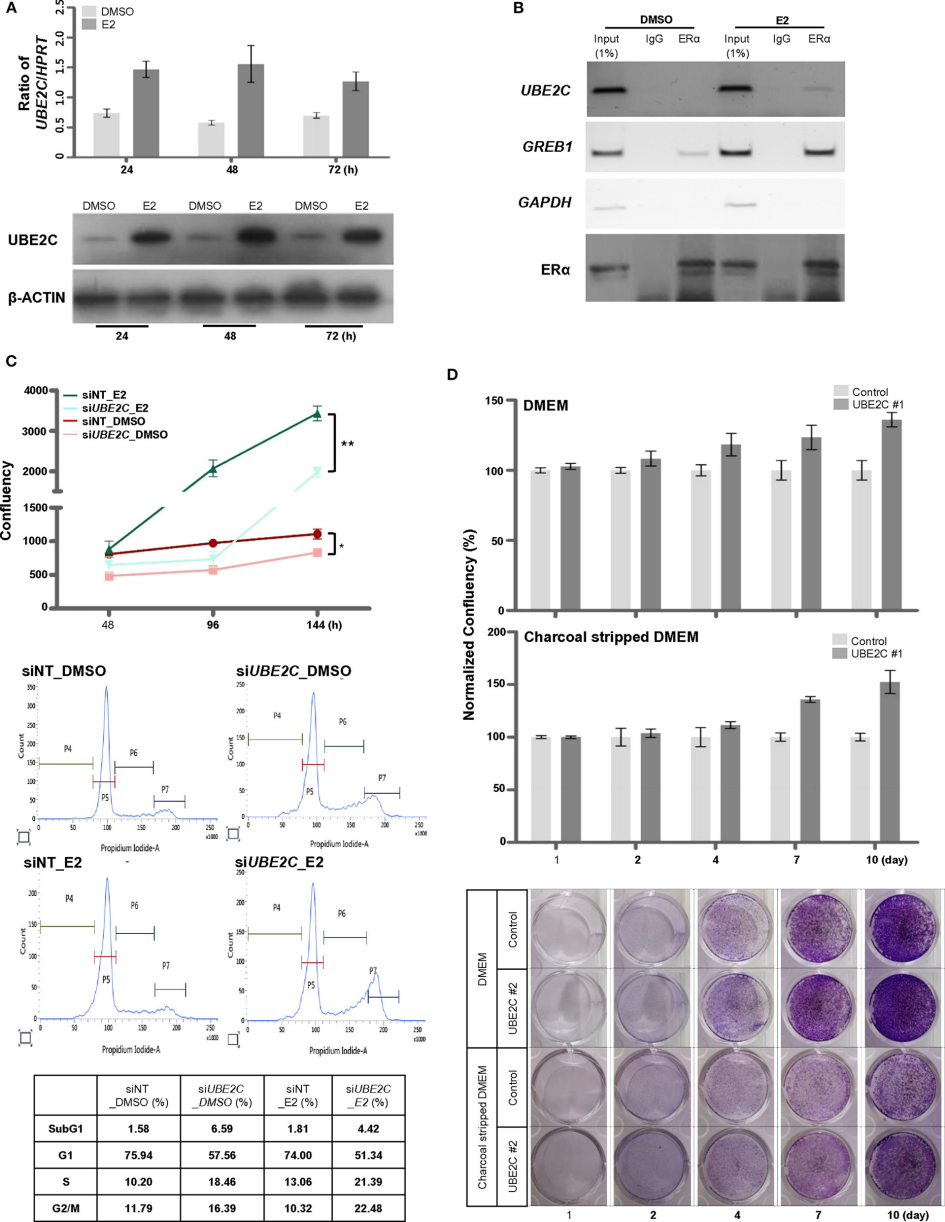
UBE2C is a target of ERα and is required for estrogen-induced cell proliferation. **(A)** The induction of *UBE2C* mRNA (upper panel) and protein (lower panel) expression by estrogen in MCF-7 cells was quantified by qRT-PCR and immunoblotting, respectively. **(B)** The ERα-bound *UBE2C* promoter region was assayed by conventional ChIP-PCR. The *GREB1* and *GAPDH* promoters served as ER α-bound positive and negative controls, respectively. **(C)** MCF-7 cells were transfected with UBE2C siRNAs for 48 h, and estrogen was added to the medium at 48 h intervals. Cell proliferation was evaluated using an IncuCyte analyzer (upper panel). MCF-7 cells were transfected with UBE2C siRNAs for 48 h, and estrogen was added to the medium for 24 h. Then, cells were stained with propidium iodide, and DNA content and intensity were analyzed by flow cytometry. **(D)** Hormone-independent cell proliferation of UBE2C-overexpressing MCF-7 cells was monitored by clonogenic assay under DMEM (upper and lower panels) or charcoal stripped DMEM (middle and lower panels) culture conditions. *n* = 3; Bars: SE. *P < 0.05, **P < 0.01, Student's *t*-test.

**Figure 5:**
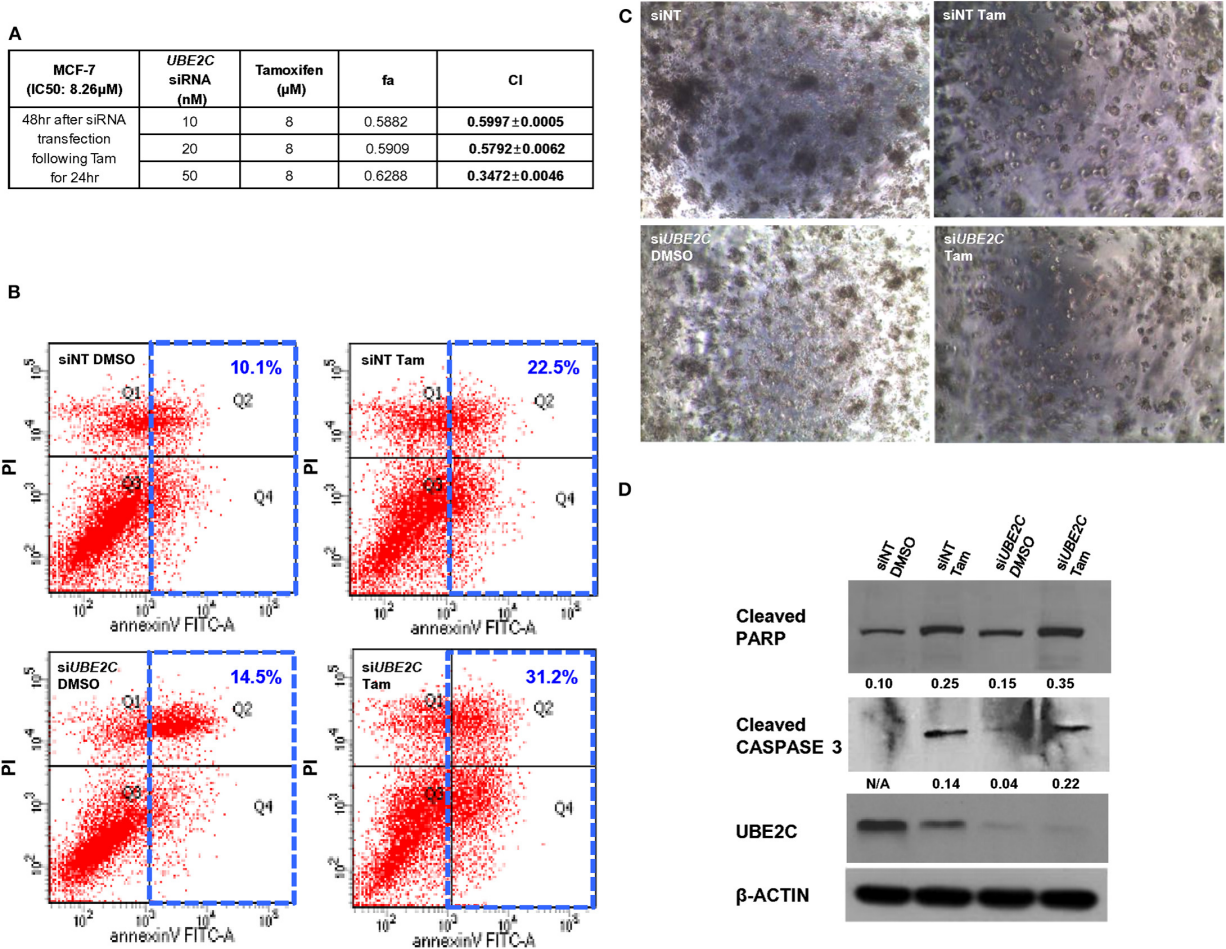
UBE2C depletion in combination with tamoxifen increases apoptosis of HR+/HER2– breast cancer cells. **(A)** Chou–Talalay drug interaction analysis in MCF-7 cells treated with *UBE2C* siRNA and tamoxifen. **(B)** The apoptotic potential of treated cells was determined by flow cytometry and Annexin V assay. The cells were stained with propidium iodide and Annexin V antibody, and DNA content and intensity were analyzed by flow cytometry. Numbers indicate the percentage of apoptotic cells. **(C)** The viability of treated MCF-7 cells was evaluated using a 3D Matrigel assay (40×). **(D)** Lysates from treated MCF-7 cells were subjected to western blotting to monitor the protein levels of cleaved PARP and cleaved CASPASE 3. Fold changes were determined by comparing the protein levels with those of β-ACTIN using ImageJ. Tam, tamoxifen.

Because tamoxifen downregulated UBE2C expression in MCF-7 cells (**Figure 5C**), we examined the effect of estrogen on UBE2C expression in HR+/HER2– breast cancer cells. MCF-7 (Figure 4A) and T47D (Supplementary Figure 1) cells were treated with E2, and UBE2C expression was monitored at the indicated time points. Exposure to E2 upregulated UBE2C at the mRNA and protein levels in MCF-7 and T47D cells.

The role of estrogen receptor α (ERα) in the regulation of *UBE2C* mRNA expression in HR+/HER2– breast cancer was evaluated by analyzing the recruitment of ERα to the promoter region of *UBE2C* in MCF-7 cells by ChIP-PCR (Figure 4B). A search of the public database (https://chip-atlas.org) was performed to predict the putative binding region of ERα in the *UBE2C* promoter, and specific sequences were selected (Supplementary Figure 2). ChIP analysis revealed increased ERα binding to *UBE2C* and *GREB1*, an ERα target gene, but not to *GAPDH*, a negative ERα target gene, in the presence of estrogen.

Given our observations suggesting direct regulation of *UBE2C* mRNA expression by ERα binding, we assessed the effect of UBE2C on cell proliferation in response to estrogen. MCF-7 cells were transfected with *UBE2C* siRNA and treated with E2, and cell growth and cell cycle progression were assessed by live cell imaging and flow cytometry, respectively (Figure 4C). Cell proliferation was significantly lower in UBE2C knockdown cells exposed to estrogen than in those cultured in the absence of estrogen. Cell cycle analysis showed that UBE2C depletion caused cell cycle arrest at the S and G2/M phases of the cell cycle, consistent with previous studies by Shen et al. (29) and Palumbo et al. (20). UBE2C knockdown strongly induced arrest at S and G2/M phases in MCF-7 cells exposed to estrogen compared with UBE2C knockdown in cells not exposed to estrogen. Taken together, these results show that loss of UBE2C inhibits cell proliferation by affecting cell cycle progression in HR+/HER2– breast cancer cells treated with estrogen.

The tumorigenicity of UBE2C led us to hypothesize that sustained UBE2C overexpression promotes estrogen-independent tumor growth. The proliferation rate of UBE2C-overexpressing MCF-7 cells cultured in estrogen-deprived medium was markedly higher than that of cells cultured in DMEM for a long period (Figure 4D). These data suggest that UBE2C overexpression contributes to estrogen-independent growth in HR+/HER2– breast cancer cells.

### Effects of UBE2C Depletion in Combination With Tamoxifen on Apoptosis of HR+/HER2– Breast Cancer Cells

To evaluate the effect of UBE2C depletion in combination with pharmacological inhibition with tamoxifen, MCF-7 (Figure 5A) and T47D (Supplementary Figure 3) cells were transfected with *UBE2C* siRNA and treated with tamoxifen, and cell growth/viability was measured. Tamoxifen inhibited the growth of HR+/HER2– breast cancer cells, with inhibitory concentration (IC_50_) values of 8.26 μM and 7.61 μM in MCF-7 and T47D cells, respectively. Next, MCF-7 and T47D cells were transfected with various concentrations of *UBE2C* siRNA, followed by tamoxifen treatment at a dose corresponding to the IC_50_ value. The Chou–Talalay method was used to assess the synergistic effect of tamoxifen and *UBE2C* siRNA. The CI was 0.3472 ± 0.0046 in MCF-7 cells and 0.8200 ± 0.0083 in T47D cells, demonstrating the synergistic effect of tamoxifen and *UBE2C* siRNA. MCF-7 cells were more sensitive to combination treatment with tamoxifen and *UBE2C* siRNA than were T47D cells. These data suggest that tamoxifen acts synergistically with UBE2C depletion to decrease cell viability in HR+/HER2– breast cancer cells expressing UBE2C.

To assess the effect of tamoxifen and UBE2C depletion on cell death, MCF-7 cells were transfected with *UBE2C* siRNA and treated with tamoxifen, and cell death was analyzed by flow cytometry (Figure 5B), 3D cell culture (Figure 5C), and immunoblotting (Figure 5D). *UBE2C* siRNA alone slightly increased the population of apoptotic cells compared with that in cells treated with non-targeting control siRNA, and tamoxifen markedly increased the population of apoptotic cells compared with the effect of *UBE2C* siRNA. Combination treatment with *UBE2C* siRNA and tamoxifen significantly increased apoptosis. The size and number of cell colonies was smaller in cells treated with *UBE2C* siRNA and tamoxifen than in those exposed to *UBE2C* siRNA alone or tamoxifen alone. The expression of cleaved PARP and cleaved CASPASE 3 was higher in cells treated with *UBE2C* siRNA and tamoxifen than in those exposed to *UBE2C* siRNA alone or tamoxifen alone. These findings suggest that UBE2C depletion markedly increased the cytotoxicity of tamoxifen by inducing apoptosis in HR+/HER2– breast cancer cells.

## Discussion

Many studies report that high UBE2C expression is associated with poor survival in patients with breast cancer irrespective of subtype (22, 24, 25, 30, 31). However, we previously showed that high *UBE2C* mRNA expression is significantly associated with poor DFS, DMFS, and OS only in patients with HR+/HER2– breast cancer among the four subtypes (10). This finding indicated that the association between *UBE2C* overexpression and the survival of patients with HR+/HER2– breast cancer might be estrogen related; thus, in this study, we assessed the effect of estrogen on UBE2C expression using HR+/HER2– breast cancer cells.

A literature review was performed to determine the correlation between the mRNA and protein expression of UBE2C. The results showed a correlation value of UBE2C mRNA-to-protein of 0.7127 (32), which is considered acceptable. Although the expression of UBE2C was only measured at the mRNA level and not at the protein level, we further assessed the clinical significance of UBE2C overexpression in HR+/HER2– breast cancer. The results of univariate and multivariate analyses showed that high *UBE2C* mRNA expression was strongly associated with poor DFS, DMFS, and OS only in patients with HR+/HER2– pN0 and pN1 (early) breast cancer but not in those with pN2/N3 (late) breast cancer. However, *UBE2C* mRNA expression was lower in pN0 and pN1 tumors from HR+/HER2– breast cancer patients than in tumors from other subtype patients. These findings indicate that the effect of *UBE2C* overexpression on the survival of patients with early HR+/HER2– breast cancer may be estrogen related, and *UBE2C* expression can predict the risk of distant recurrence among patients with early HR+/HER2– breast cancer. In addition, *UBE2C* mRNA expression was not associated with clinical outcomes in HR+/HER2– pN2/N3 breast cancer and other more aggressive subtypes (HR+/HER2+, HR–/HER2+, and TNBC). This finding suggests that although UBE2C expression correlates with worse prognosis of early HR+/HER2– breast cancer in this study, since most highly malignant breast cancer cases also show high UBE2C expression, it implies that the expression does not hold a prognostic value.

Consistent with the results of two studies by Takeo et al. and Chou et al. in MCF-7 cells (23, 33), functional *in vitro* experiments based on UBE2C overexpression or depletion showed that aberrantly high levels of UBE2C promoted cell proliferation and migration. These previous studies were based on literature reviews on the importance of UBE2C tumorigenicity as an essential part of the ubiquitin-conjugating enzyme complex, and they used the MCF-7 cell line as a representative breast cancer cell line. However, this study analyzed the association between *UBE2C* expression and clinical outcomes only in HR+/HER2– breast cancer through a parametric survival analysis using public datasets and a patient cohort. The MCF-7 cell line was used as a representative HR+/HER2– breast cancer cell line. Although MCF-7 cells are widely used in research, cancer cell lines are different from patient-derived cancer cells. We therefore confirmed the results obtained in MCF-7 cells by using a 3D cell culture system.

Estrogen is a key regulator of female development and reproductive function, and it is a causal factor in breast cancer. Estrogen-driven gene expression is mediated by the activity of ERα in normal breast cells and in breast cancer (34). We found that estrogen exposure highly upregulated UBE2C mRNA and protein, and that ERα directly bound to the *UBE2C* promoter region. Loss of UBE2C expression markedly decreased cell proliferation by affecting cell cycle progression under estrogen exposure conditions compared with UBE2C knockdown cells without estrogen exposure in HR+/HER2– breast cancer cells. These findings suggest that UBE2C is a target of ERα and is required for estrogen-induced cell proliferation, thereby activating estrogen-mediated signaling networks. This may explain why high *UBE2C* expression was strongly associated with poor DMFS, DFS, and OS only in patients with HR+/HER2– breast cancer. In addition, we found that *UBE2C* mRNA expression levels were high in certain triple negative cell lines. Nicolau-Neto et al. reported that UBE2C is a transcriptional target of the cell cycle regulator FOXM1, which was identified as a specific marker for TNBC (35, 36).

Tamoxifen is currently used for the management of ER+ breast cancers. We found that tamoxifen increased the apoptotic population of MCF-7 cells. The cytotoxicity of tamoxifen is attributed to the induction of apoptosis in breast cancer cells (37, 38). Despite its antitumor efficacy in the clinic, 20–30% of patients with ER+ breast cancer are resistant to tamoxifen treatment (39). However, the mechanisms underlying resistance to tamoxifen treatment are complex and not fully understood (40–42). Dysregulation of apoptosis or cell cycle regulators is suggested as one of several mechanisms of resistance to endocrine therapy (41). In this study, we showed that UBE2C is involved in cell cycle regulation, and that UBE2C overexpression contributes to estrogen-independent growth in HR+/HER2– breast cancer cells. The present findings indicate that UBE2C overexpression may induce resistance to tamoxifen treatment by promoting estrogen-independent growth. UBE2C depletion markedly increased the cytotoxicity of tamoxifen by inducing apoptosis in HR+/HER2– breast cancer cells. This suggests that tamoxifen treatment combined with inhibitors targeting UBE2C could be an effective strategy to increase the inhibitory effect of tamoxifen and overcome resistance in HR+/HER2– breast cancer. The combination of palbociclib, a selective inhibitor of the cyclin-dependent kinases CDK4 and CDK6, and ovarian suppression with letrozole significantly prolonged progression-free survival in women with ER+/HER2– advanced breast cancer in the PALOMA-3 (NCT01942135) study (43). Therefore, we suggest that the combination of palbociclib with tamoxifen is a promising treatment strategy in patients with HR+/HER2– breast cancer overexpressing UBE2C.

## Data Availability Statement

All datasets generated for this study are included in the article/Supplementary Material.

## Ethics Statement

The studies involving human participants were reviewed and approved by the Samsung Medical Center (SMC) Institutional Review Board (IRB file No. 2008-12-035). Written informed consent for participation was not required for this study in accordance with the national legislation and the institutional requirements.

## Author Contributions

Y-JK and YS: conception and design and final approval of the article. Y-JK, GL, JH, KS, J-SC, Y-LC, and YS: analysis and interpretation. Y-JK, GL, and JH: data collection. Y-JK and JH: writing the article.

### Conflict of Interest

JH was employed by the company *Gencurix, Inc*. The remaining authors declare that the research was conducted in the absence of any commercial or financial relationships that could be construed as a potential conflict of interest.

## Supplementary Material

Supplementary Figure 1 UBE2C is induced by estrogen in T47D cells. The induction of UBE2C mRNA (left panel) and protein (right panel) expression by estrogen in T47D cells was quantified by qRT-PCR and immunoblotting, respectively. Fold changes were determined by comparing the protein levels with those of β-ACTIN using ImageJ.

Supplementary Figure 2 The putative binding region of ERα on the *UBE2C* promoter was determined by a review of https://chip-atlas.org. **(A)** A binding region of ERα on the promoter from the SRX1531791 ChIP-sequencing dataset using Integrative Genomics Viewer. **(B)** Design of the putative *UBE2C* promoter region ChIP-PCR primer pair.

Supplementary Figure 3 Chou–Talalay drug interaction analysis of UBE2C siRNA and tamoxifen in T47D cells.

Supplementary Table 1 Univariate analysis of the effect of *UBE2C* mRNA expression on disease-free survival (DFS), distant metastasis-free survival (DMFS), and overall survival (OS) in patients with HR+/HER2– breast cancer.

## Abbreviations

ER: Estrogen receptor
PR: Progesterone receptor
HER2: Human epidermal growth factor receptor 2
FFPE: Formalin-fixed paraffin-embedded
DMFS: Distant metastasis-free survival
DFS: Disease-free survival
OS: Overall survival.
