# Supplementary material for: UBE2C Overexpression Aggravates Patient Outcome by Promoting Estrogen-Dependent/Independent Cell Proliferation in Early Hormone Receptor-Positive and HER2-Negative Breast Cancer
Source: Front Oncol. 2020 Jan 23;9:1574. doi: 10.3389/fonc.2019.01574 (PMC6989552; doi:10.3389/fonc.2019.01574)
Supplement: Supplementary Figure 1 — UBE2C is induced by estrogen in T47D cells. The induction of UBE2C mRNA (left panel) and protein (right panel) expression by estrogen in T47D cells was quantified by qRT-PCR and immunoblotting, respectively. Fold changes were determined by comparing the protein levels with those of β-ACTIN using ImageJ. [file Presentation_1.PPTX]

## Slide 1
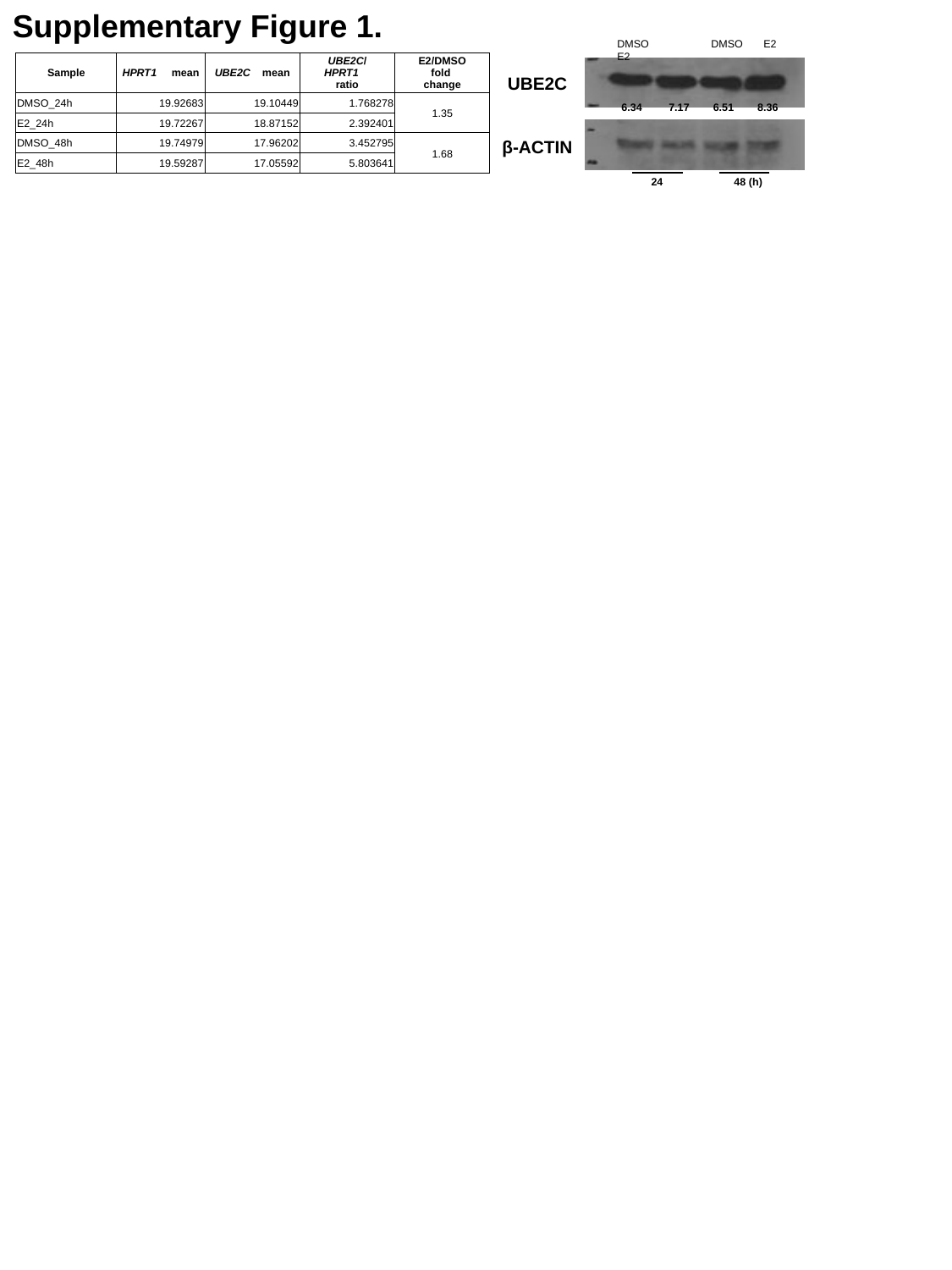

Supplementary Figure 1.
DMSO E2
DMSO E2
| Sample | HPRT1 mean | UBE2C mean | UBE2C/ HPRT1 ratio | E2/DMSO fold change |
| --- | --- | --- | --- | --- |
| DMSO\_24h | 19.92683 | 19.10449 | 1.768278 | 1.35 |
| E2\_24h | 19.72267 | 18.87152 | 2.392401 | |
| DMSO\_48h | 19.74979 | 17.96202 | 3.452795 | 1.68 |
| E2\_48h | 19.59287 | 17.05592 | 5.803641 | |
UBE2C
 6.34 7.17 6.51 8.36
β-ACTIN
24 48 (h)

## Slide 2
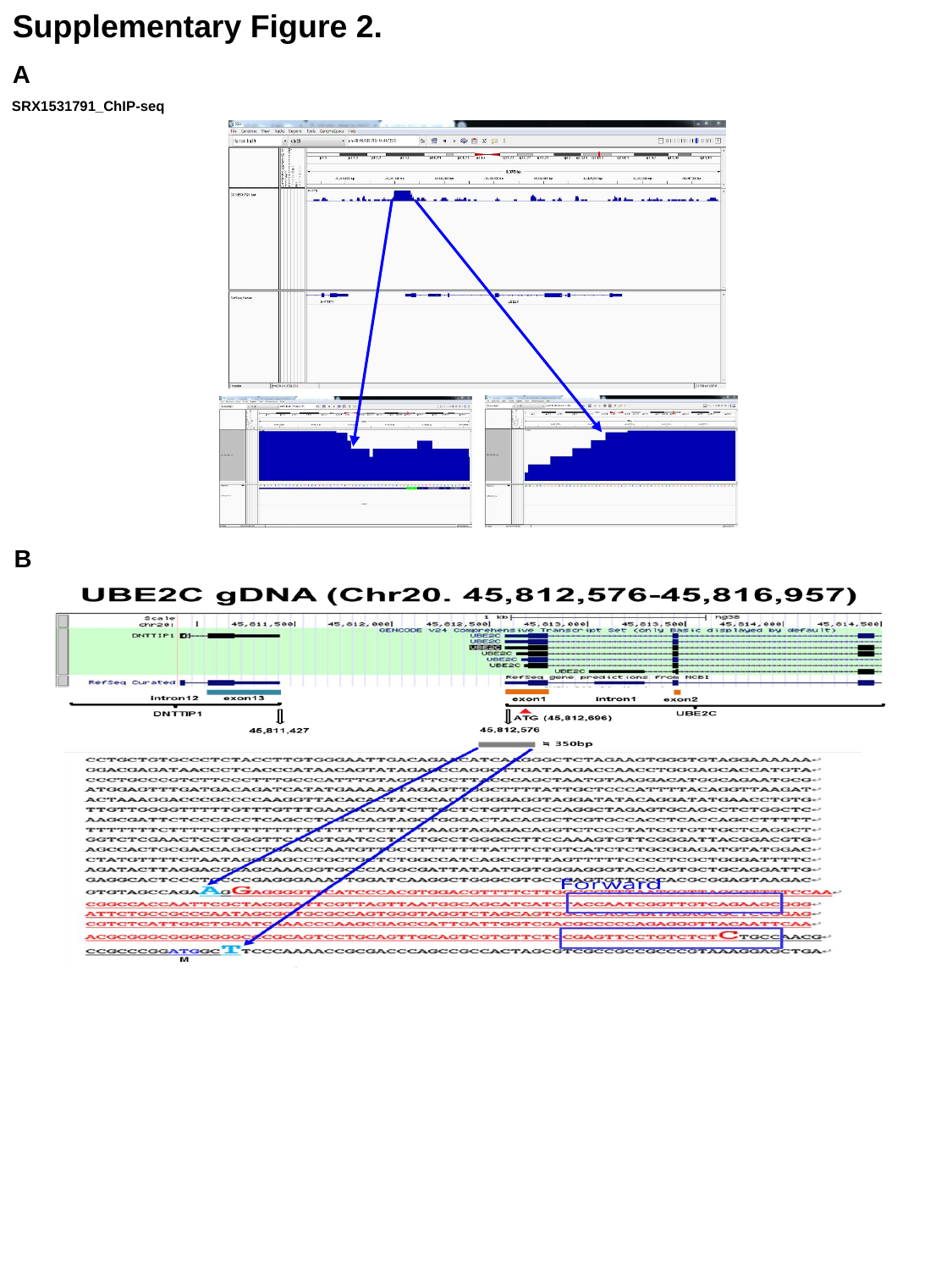

Supplementary Figure 2.
A
SRX1531791_ChIP-seq
B

## Slide 3
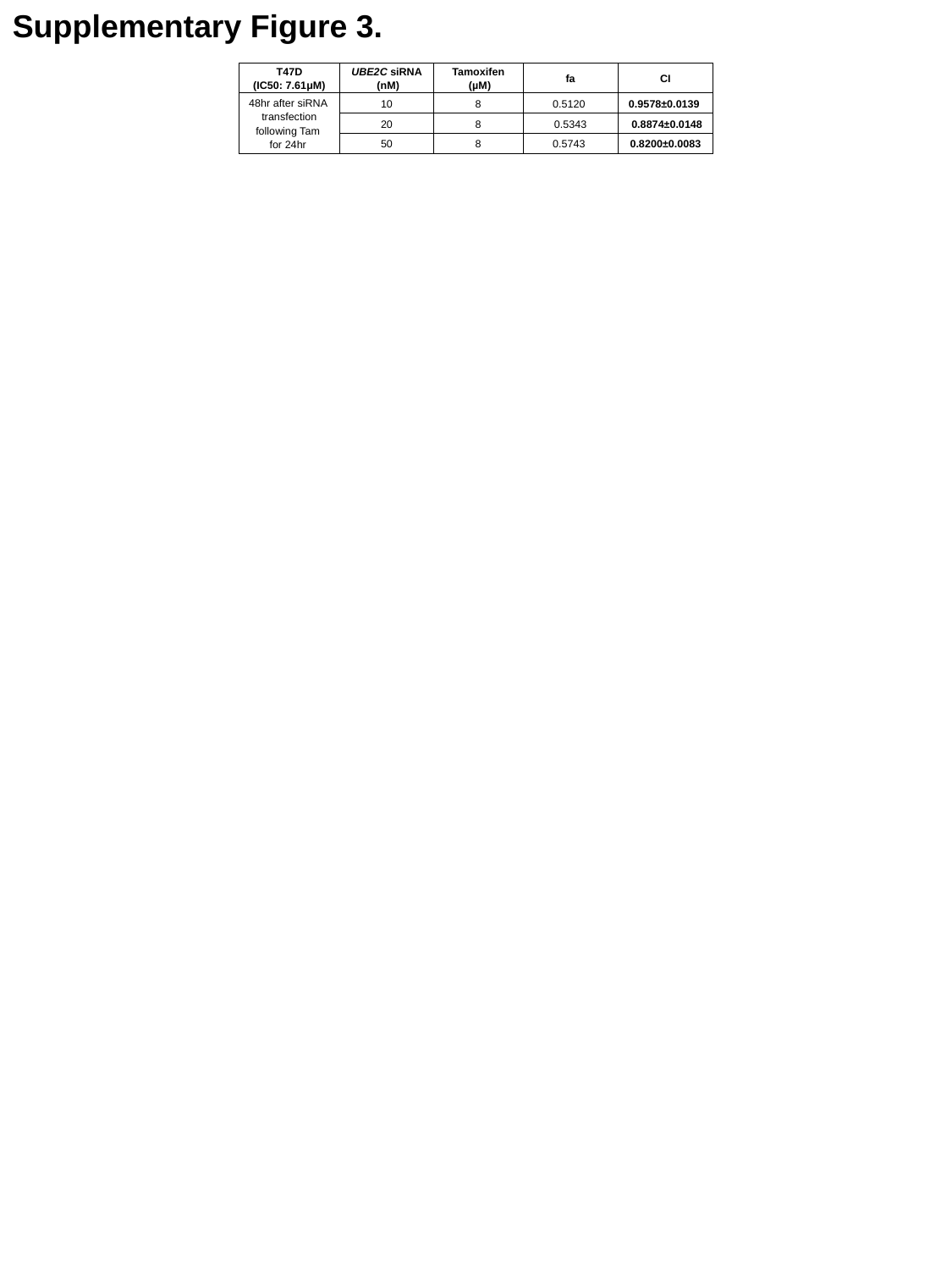

Supplementary Figure 3.
| T47D (IC50: 7.61μM) | UBE2C siRNA (nM) | Tamoxifen (µM) | fa | CI |
| --- | --- | --- | --- | --- |
| 48hr after siRNA transfection following Tam for 24hr | 10 | 8 | 0.5120 | 0.9578±0.0139 |
| | 20 | 8 | 0.5343 | 0.8874±0.0148 |
| | 50 | 8 | 0.5743 | 0.8200±0.0083 |
