# Supplementary material for: UBE2C Overexpression Aggravates Patient Outcome by Promoting Estrogen-Dependent/Independent Cell Proliferation in Early Hormone Receptor-Positive and HER2-Negative Breast Cancer
Source: Front Oncol. 2020 Jan 23;9:1574. doi: 10.3389/fonc.2019.01574 (PMC6989552; doi:10.3389/fonc.2019.01574)
Supplement: Supplementary Table 1 — Univariate analysis of the effect of UBE2C mRNA expression on disease-free survival (DFS), distant metastasis-free survival (DMFS), and overall survival (OS) in patients with HR+/HER2– breast cancer. [file Table_1.DOCX]

**Supplementary Table 1.** Univariate analysis of *UBE2C* mRNA expression for disease-free survival (DFS), distant metastasis-free survival (DMFS), and overall survival (OS) according in patients with HR+/HER2- breast cancer

|  |  | DFS | | |  | DMFS | | |  | OS | | |
| --- | --- | --- | --- | --- | --- | --- | --- | --- | --- | --- | --- | --- |
|  |  | Hazard Ratio | 95% C.I. | *P value* |  | Hazard Ratio | 95% C.I. | *P value* |  | Hazard Ratio | 95% C.I. | *P value* |
| **All** |  |  |  |  |  |  |  |  |  |  |  |  |
| GeneExpression |  |  |  |  |  |  |  |  |  |  |  |  |
| *UBE2C* | (*Low vs. High*) | 2.48 | 1.62-3.78 | **<0.001** |  | 2.31 | 1.49-3.59 | **<0.001** |  | 2.79 | 1.68-4.61 | **<0.001** |
| Clinical variable |  |  |  |  |  |  |  |  |  |  |  |  |
| pN status | (*pN0 vs. pN1*) | 2.19 | 1.38-3.46 | **<0.001** |  | 2.48 | 1.51-4.05 | **<0.001** |  | 2.60 | 1.52-4.42 | **<0.001** |
|  | (*pN0 vs. pN2&3*) | 3.25 | 2.10-5.04 | **<0.001** |  | 3.73 | 2.33-5.97 | **<0.001** |  | 3.62 | 2.18-6.02 | **<0.001** |
| Tumor size | (*≤2cm vs. >2cm*) | 1.66 | 1.14-2.43 | **0.008** |  | 1.66 | 1.11-2.46 | **0.013** |  | 1.64 | 1.07-2.51 | **0.024** |
| Histologic grade | (*I&II vs. III*) | 1.58 | 1.08-2.30 | **0.017** |  | 1.66 | 1.12-2.47 | **0.012** |  | 1.96 | 1.29-2.98 | **0.002** |
| **pN0** |  |  |  |  |  |  |  |  |  |  |  |  |
| Gene Expression |  |  |  |  |  |  |  |  |  |  |  |  |
| *UBE2C* | (*Low vs. High*) | 3.27 | 1.43-7.48 | **0.005** |  | 2.95 | 1.20-7.24 | **0.019** |  | 5.03 | 1.50-16.87 | **0.009** |
| Clinical variable |  |  |  |  |  |  |  |  |  |  |  |  |
| Tumor size | (*≤2cm vs. >2cm*) | 1.44 | 0.74-2.79 | 0.285 |  | 1.44 | 0.70-2.99 | 0.323 |  | 1.91 | 0.85-4.30 | 0.119 |
| Histologic grade | (*I&II vs. III*) | 1.24 | 0.60-2.59 | 0.562 |  | 1.69 | 0.79-3.64 | 0.180 |  | 2.66 | 1.19-5.95 | **0.017** |
| **pN1** |  |  |  |  |  |  |  |  |  |  |  |  |
| Gene Expression |  |  |  |  |  |  |  |  |  |  |  |  |
| UBE2C | (*Low vs. High*) | 2.77 | 1.31-5.85 | **0.008** |  | 2.52 | 1.18-5.38 | **0.017** |  | 3.59 | 1.47-8.77 | **0.005** |
| Clinical variable |  |  |  |  |  |  |  |  |  |  |  |  |
| Tumor size | (*≤2cm vs. >2cm*) | 1.32 | 0.69-2.54 | 0.400 |  | 1.23 | 0.63-2.43 | 0.545 |  | 0.85 | 0.42-1.72 | 0.654 |
| Histologic grade | (*I&II vs. III*) | 1.19 | 0.61-2.29 | 0.613 |  | 1.09 | 0.54-2.19 | 0.816 |  | 1.21 | 0.58-2.52 | 0.620 |
| **pN2/N3** |  |  |  |  |  |  |  |  |  |  |  |  |
| Gene Expression |  |  |  |  |  |  |  |  |  |  |  |  |
| *UBE2C* | (*Low vs. High*) | 1.51 | 0.78-2.92 | 0.219 |  | 1.55 | 0.78-3.08 | 0.209 |  | 1.47 | 0.71-3.01 | 0.298 |
| Clinical variable |  |  |  |  |  |  |  |  |  |  |  |  |
| Tumor size | (*≤2cm vs. >2cm*) | 0.87 | 0.42-1.81 | 0.716 |  | 0.79 | 0.38-1.65 | 0.537 |  | 1.05 | 0.46-2.37 | 0.915 |
| Histologic grade | (*I&II vs. III*) | 1.57 | 0.85-2.93 | 0.152 |  | 1.50 | 0.79-2.87 | 0.215 |  | 1.68 | 0.85-3.30 | 0.133 |

pN, lymph node status; CI, confidence interval.
